# Barriers to using clozapine in treatment-resistant schizophrenia: systematic review

**DOI:** 10.1192/bjb.2018.67

**Published:** 2019-02

**Authors:** Saeed Farooq, Abid Choudry, Dan Cohen, Farooq Naeem, Muhammad Ayub

**Affiliations:** 1 Keele University, UK; 2 Birmingham Women's and Children's NHS Foundation Trust, UK; 3 Mental Health Organization North-Holland North Heerhugowaard, The Netherlands; 4 Queen's University, Kingston, Canada

**Keywords:** Treatment resistant schizophrenia, clozapine, barriers

## Abstract

**Aims and method:**

To systematically review the literature on barriers to the use of clozapine and identify any interventions for optimizing clozapine use in treatment-resistant schizophrenia. Journal databases were searched from 1972 to March 2018. The following search terms were used: treatment-resistant schizophrenia, clozapine, barriers, use, prescription rates, implementation, clozaril and prescribing practices. Following a review of the literature, 15 papers were included in the review.

**Results:**

The major barriers that were identified included mandatory blood testing, fear of serious side-effects and lack of adherence by the patients, difficulty in identifying suitable patients, service fragmentation, and inadequate training in or exposure to using clozapine.

**Clinical implications:**

In view of consistent evidence across the studies on inadequate knowledge and skills as a significant barrier, we suggest that a certification requiring competence in initiating and managing side-effects of clozapine becomes a mandatory requirement in training programmes.

**Declarations of interest:**

None.

## Clozapine use in schizophrenia

Clozapine is the only medication licensed for treatment-resistant schizophrenia (TRS), which affects about one-third of those suffering from the disorder. Recently, there has been increased interest in redefining the role of clozapine in the treatment of schizophrenia in view of the evidence of superior efficacy and safety, despite serious side-effects.^1^ Meta-analyses have demonstrated that clozapine is significantly better at treating symptoms than first-generation antipsychotics and some (but not all) second-generation antipsychotics.^2^ This superior efficacy was also supported by two large, independently funded studies.^3^^,^^4^ Clozapine also appears to have broader effects, with evidence for efficacy in suicidality, aggression and substance misuse.^1^ In the USA, clozapine is approved by the Food and Drug Administration for the management of suicidality in people with schizophrenia or schizoaffective disorder. In addition, clozapine has been shown to have anti-aggressive properties^5^ and may also be effective in diminishing substance misuse.^6^^,^^7^ Tiihonen *et al*^7^ found, using a large database, that that people regularly taking clozapine had the lowest risk of premature mortality compared with both those on other antipsychotics and those not taking regular medication, despite the fact that the drug is associated with a number of serious adverse side-effects.^7^

## Potential barriers and delays in clozapine use

Despite the evidence of superior efficacy and recommendations by different treatment guidelines, the drug is grossly underutilised.^8^ Studies based on prescription patterns in routine practice almost universally show lower prescriptions of clozapine in individuals with Schizophrenia, even after taking into account potential barriers such as inadequate service provision.^9^ There is also substantial evidence that the use of clozapine is delayed for several years, which may result in less than optimal efficacy for the drug. A study by Howes *et al*^10^ showed that the mean theoretical delay from meeting the National Institute for Health and Care Excellence (NICE) criteria for TRS and starting clozapine was about 4 years. In New Zealand the theoretical delay was almost 10 years.^11^ In the USA, only six states reported that more than 10% of Medicaid-eligible patients with schizophrenia had received a prescription of clozapine.^12^

The reasons for such suboptimal use of clozapine remain obscure, and may include several factors related to patients, carers and clinicians. These may include the perception of the drug as a dangerous medicine^1^ or difficulties associated with initiating and maintaining the treatment. The life-threatening side-effects of clozapine and mandatory requirement for white blood cell (WBC) counts may partly account for the less than optimal use of the drug in clinical practice. Experience in using clozapine may be an important factor. A study by Nielsen *et al*^13^ of the attitudes and knowledge of 137 psychiatrists in Denmark, including 100 consultant psychiatrists, revealed that some had never prescribed clozapine despite having worked for over 5 years. The barriers to effective use of clozapine have not been reviewed systematically.

We therefore aimed to review the literature on barriers to the effective use of clozapine in clinical practice for TRS. We also wanted to identify any interventions that could potentially improve the use of clozapine. This systematic review aimed to answer the following questions.

• What barriers or factors have been identified that prevent the optimal use of clozapine in TRS, based on the current literature?
• What strategies have been explored to promote the effective use of clozapine in TRS?
• What is the methodological quality of the evidence that is available exploring the barriers to optimal use of clozapine?

## Method

We followed the Preferred Reporting Items for Systematic Reviews and Meta-Analyses (PRISMA) statement guidelines.^14^ A protocol defining the key methodological parameters was developed prior to the literature search and was registered at the International Prospective Register of Systematic Reviews (PROSPERO).^15^

### Search strategy

Electronic databases (PsycINFO, Medline, PubMed, AMED, CINHAL and EMBASE) from 1972 onwards were searched, followed by a search of the reference lists of the full texts of the retrieved articles for further relevant articles. The following search terms were used: treatment-resistant schizophrenia, clozapine, barriers, use, prescription rates, implementation, clozaril and prescribing practices. These keywords were searched for in the title, keywords, or abstract. Truncations and related terms were used as appropriate based on individual database procedures. The search was last updated in March 2018.

All study types (intervention, observational and descriptive) were included in the review if the following inclusion criteria were met.

• Adult populations with a diagnosis of TRS for whatever indication. Clozapine has been used for other diagnoses; however, we limited our present review to TRS.
• Included primary research information on the outcome variables, i.e. barriers or factors associated with low use or implementation strategies.
• Published between 1972 and 2018.

Studies that examined the pattern of use of clozapine, the rate of prescriptions, or its efficacy and effectiveness were excluded, unless these provided data on the barriers or factors associated with low or high use of clozapine.

There is no agreed definition of the ‘optimal use’ of clozapine. However, a number of studies^9^^–^^11^^,^^16^ indicate that the optimal use is determined on the basis of time since the start of the first antipsychotics (considering that clozapine is used after failure to respond to two antipsychotics) and the prevalence of clozapine prescription relative to total antipsychotic prescriptions (based on fact that about 30% of those suffering from schizophrenia develop TRS). These provide useful guidance but do not take into practical factors such as patient willingness to start clozapine or non-availability, or the cost of clozapine in low- and middle-income country settings. We used these parameters as a broad guideline for our review, but we will also report clozapine use and how it is defined as adequate or optimal by different studies.

### Data extraction

The screening for searches examining the relevant abstracts, examination of full-text articles and data extraction were done by two reviewers independently, as outlined in the protocol.^15^ Any disagreements were resolved by consensus and, where appropriate, by consultation with the third reviewer. A data extraction sheet was developed based on the pre-specified outcomes and relevant data were extracted on to this sheet. We planned a meta-analysis of primary and secondary outcomes, but it was not possible to statistically summarise the data owing to the heterogeneity of studies, lack of adequate data and low quality of studies. We instead provide a descriptive summary of main findings.

## Results

**Fig. 1:**
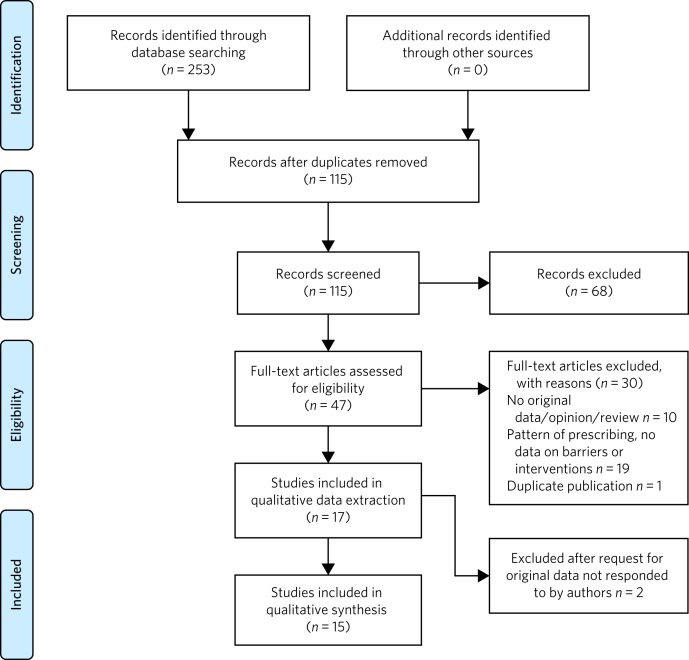
Summary of the abstracts reviewed to identify papers relevant for the review.

The electronic searches returned 253 relevant abstracts and titles; no further articles were identified from the other sources. We screened the titles and abstracts, and excluded any studies that were not directly relevant to the objectives of the review. After screening these titles and abstracts and removal of duplicates, we further examined 47 full-text papers. Finally, we included 15 papers in the review. The details of the search yield and reasons for excluding full-text articles are provided in Fig. 1.

### Characteristics of included studies

The studies were conducted in a number of different populations, settings and periods, and also used diverse methodologies. Owing to the diverse methodologies and number of variables examined across studies, it was inappropriate to pool the data to produce a statistical summary. We therefore describe the main findings and produce a narrative summary of results.

Fifteen studies met the inclusion criteria. Twelve of these studies focused on barriers or factors associated with clozapine use. These comprised surveys (*n* = 5), case note reviews (*n* = 4), and semi/structured interviews and consultations with stakeholders (*n* = 3). The majority of these studies (*n* = 8) involved eliciting views from clinicians, particularly consultant psychiatrists. Three studies described interventions or quality improvement initiatives to facilitate the use of clozapine. These are described separately.

In survey-based studies, response rates varied from 8.8 to 76%. The mean response rate from the papers which had figures available (*n* = 5) was 52.3%. The total number of males from the studies providing this information (*n* = 6) was 608, and the number of females was 402. The populations in these studies comprised 902 psychiatrists, 68 trainees, 49 pharmacy staff and 15 nursing staff or staff in mental health leadership positions. One database study reviewed the Medicaid patients on antipsychotic medication using records of 629 800 patients in the analysis.

In the three intervention studies, 158 participants were involved. One study did not provide details of sample size.

The characteristics of included studies are shown in Table 1.

**Table 1:** Characteristic of included studies

Author/year	Population	Method/design	Sample characteristics and response rates (ReR)
1. Gees *et al* (2013)	All staff at South London and Maudsley NHS Foundation Trust	Survey	*n* = 144 Trainee doctors 42% *n* = 60 Consultants 14% *n* = 20 Pharmacy staff 16% *n* = 23
2. Cirulli (2005)^24^	Consultant psychiatrists working in child and adolescent mental health services in-patient unit	Survey	*n* = 83 ReR 59 (71%)
3. Najim *et al* (2013)^20^	Out-patients on clozapine in UK community population	Retrospective case note review	*n* = 42 ReR 100%
4. Swinton & Ahmed (1999)^19^	In-patients in high-secure hospital – patients, consultants, nurses	Case note review and survey of patients and staff	*n* = 95 ReR 72 (76%)
5. Grover *et al* (2015)^21^	Patients initiated on clozapine in North India tertiary hospital (Jan 2006–June 2014)	Retrospective record review	*n* = 200 patients
6. Tungaraza & Farooq (2015)^22^	Psychiatrists	Survey	*n* = 2771 ReR 243 (8.8%)
7. Apiquian *et al* (2004)^23^	Psychiatrists	Survey	*n* = 200 ReR - 148 (74%)
8. Goren *et al* (2016)^26^	Key informants (Psychiatrists, clinical pharmacists, advanced practice nurses) involved in the clozapine process at US Department of Veteran Affairs with high and low utilization of clozapine	Semi structured telephone interviews	*n* = 70 participants Psychiatrist 31.4% Pharmacy staff 37.1% Mental health leadership 15.7% Advanced practice nurse 5.7% Other 10%
9. Kelly *et al* (2015)^18^	Psychiatry residents, fellows, and psychiatrists in the state of Maryland	Survey with each question rated using Likert scale: 1 = strongly disagree, 5 = strongly agree	*n* = 860 ReR 277 (32%)
10. Stroup *et al* (2014)^25^	Patients with schizophrenia spectrum disorder, using Medicaid data from 2001 to 2005, who used clozapine or standard antipsychotic medication in one or more treatment episodes	Comparison between standard antipsychotic and clozapine use, using statistical analysis	*n* = Patients on clozapine (*n* = 15 524) Patients on other antipsychotics (*n* = 614 285)
11. Nielson *et al* (2009)^13^	Psychiatrists from six counties in Denmark; three highest and three lowest prescription rates of clozapine	Structured interview	*n* = 100 72 Consultant psychiatrists 20 psychiatrists 8 trainee psychiatrists
12. Kelly *et al* (2018)^27^	Clinicians and researchers identified by the National Association of State Mental Health Program Directors	Expert opinion, literature review and focus group	11 Psychiatrists and researchers; however, no specific details given
Intervention studies			
13. Carruthers *et al* (2016)^28^	Academics and clinicians in clozapine prescribing and patients with treatment-resistant schizophrenia in receipt of Medicaid in New York	Educational initiative utilizing web-based modules to educate consumers and carers as well as clinicians regarding clozapine use	No sample details provided
14. Nielson *et al* (2012)^13^	Psychiatric out-patients on treatment with clozapine in Denmark	Point-of-care (POC) testing using capillary sampling *v.* venous sampling	85 participants
15. Bogers *et al* (2015)^29^	Patients established on clozapine	Randomised cross-over trial design for POC testing using capillary sampling *v.* venous sampling	73 patients were included in this study; three dropped out before completion

#### Barriers to the use of clozapine in TRS

It was possible to classify the barriers in three groups with some overlap:

• barriers related to patients and the drug;
• clinician-related barriers;
• health system-related factors.

#### Patient- and drug-related barriers

Five studies commented on patient-related factors affecting the use of clozapine in TRS. The complete refusal of blood tests was considered a major barrier, with patients delaying the initiating of clozapine (56%, *n* = 72).^17^ This was replicated by Kelly *et al*,^18^ who surveyed psychiatrists in Maryland, USA, and found that the main barrier, ranked highest on the Likert scale (1–5), was patient non-adherence with blood work (3.7 ± 1.1) and the burden of blood work on the patient (3.6 ± 1.2).^18^ In a survey of patients, Swinton and Ahmed (1999)^19^ reported that almost two-thirds of participants did not want the burden of regular blood tests. This was replicated in a survey of staff, with 65% (*n* = 83) reporting that patients did not want the burden of regular blood tests and that frequent blood tests were considered a major barrier to initiating clozapine.^17^

Concerns about tolerating clozapine were considered to be fairly or very frequently related to delays in clozapine use by 46% (*n* = 59) of clinical staff.^17^ Complications related to clozapine, such as constipation, hypersalivation, myocarditis and neutropenia, can inhibit clozapine use; a survey of clinical staff found that 37% (*n* = 76) felt that these potential medical complications frequently restricted the use of clozapine.^17^

Najim *et al*^20^ reviewed 42 case notes of patients on clozapine and found that there were significant delays in commencing clozapine in patients aged over 30.^20^ This was replicated by Grover *et al*,^21^ who carried out a case note review on 200 in-patients from a tertiary care centre in North India. A greater delay in initiating clozapine was noted in the older age group (over 20) compared with those under 20 (mean 0.91 *v.* 2.05; s.d. 1.05 *v.* 1.86).^21^ In addition, they found a significant delay in patients prescribed polypharmacy compared with non-polypharmacy (mean 2.58 *v.* 1.68; s.d. 2.06 *v.* 1.67), and delays were also associated with being in an urban locality (mean 2.11 *v.* 1.37; s.d. 1.98 *v.* 1.11).^21^

#### Clinician-related factors

Inadequate knowledge of or experience in clozapine use. Fifty-two per cent (*n* = 75) of staff surveyed in South London Maudsley NHS Foundation Trust^17^ reported not being familiar with initiation of clozapine. In another large survey, 74% (*n* = 136 total 184) of psychiatrists working in the UK also highlighted a lack of knowledge or experience amongst consultants, leading to delays.^22^ A significant number of consultants (42%; *n* = 96) had fewer than five patients on clozapine, despite half of these consultants working in trusts with a dedicated clozapine service and having been in-post for 7 years.^22^ This was replicated by Nielson *et al* (2009), who found that 48% of psychiatrists surveyed had treatment responsibility for fewer than five patients treated with clozapine.^13^ In Mexico, Apiquian *et al*^23^ reported that fewer than half of the 200 surveyed psychiatrists in Mexico knew the recommended average dose of clozapine.^23^

The fear of side-effects or lack of knowledge in dealing with these were considered to be serious hurdles in initiating clozapine. Sixty per cent (*n* = 70) of practitioners surveyed in South London and Maudsley NHS Foundation Trust raised concerns about tolerability and side-effects that delayed the initiation of clozapine.^17^ Nielson *et al*^13^ reported that in terms of side-effects and knowledge, only 33% (*n* = 33) knew that the risk of agranulocytosis was highest in the first 6 months and 23% (*n* = 23) overestimated this risk of agranulocytosis.^13^

The majority of the clinicians in a survey (78%; *n* = 105) said they would support clozapine initiation after a trial of two antipsychotics.^17^ However, Nielson *et al* (2009) found that only 44.9% (*n* = 44) would go to clozapine after two antipsychotics,^13^ and about a third 30.6% (*n* = 30)^13^ of clinicians in one survey and 14% (*n* = 19) in another would wait until three adequate trials of antipsychotics prior to initiating clozapine, while 18.4% (*n* = 19)^13^ would wait until more than three failed adequate trials of antipsychotics. In another survey, 28% (*n* = 51, total 184) of consultants said they would trial at least another antipsychotic before going to clozapine after a failed trial of two antipsychotics,^22^ and 40.5% (*n* = 92) preferred to use several other antipsychotics before clozapine.^19^ Nielson *et al*^13^ found that 64.7% of psychiatrists surveyed (*n* = 64) would rather combine two antipsychotics than prescribe clozapine, and 15.2% (*n* = 15) would augment with a mood stabiliser before using clozapine in a non-schizoaffective state.^13^

Difficulty in identifying suitable patients and unclear diagnosis were highlighted by 12% of consultant psychiatrists (*n* = 22) in a survey conducted by Tungaraza & Farooq.^22^ Although consultants felt they had good exposure to clozapine as trainees, 36.2% (*n* = 83) felt it was not easy to identify suitable patients for clozapine.^22^

##### Need for intense monitoring

Forty-two per cent (*n* = 77) of psychiatrists in a UK-wide survey felt it was complex and cumbersome to initiate and mange clozapine, which led to delays in starting the drug.^22^ In a survey of consultants based in child and adolescent psychiatry, 29% (*n* = 17) reported that they did not prescribe clozapine owing to the need for intense monitoring.^24^ Tungaraza & Farooq^22^ found that 74% (*n* = 136) of clinicians felt there were delays owing to refusal of patients to have blood tests.^22^

##### Serious side-effects

In a survey of consultant psychiatrists, 105 out of 231 respondents (45.5%) acknowledged that their patients experienced untoward side-effects while on clozapine, which was considered to be major factor in delaying clozapine use.^22^

Staff in child and adolescent services highlighted unfamiliarity with clozapine (41%; *n* = 4) and side-effects (41%; *n* = 4)^24^ as major factors in delaying clozapine initiation. Swinton & Ahmed^19^ reported that 22% (*n* = 7) of the clinical staff in their study believed that the risks associated with clozapine outweighed the benefits of starting clozapine.^19^

##### Perception that patients may not adhere to treatment

Clinical staff surveyed at a high-secure hospital reported likely poor adherence by the patients as a reason for not prescribing clozapine in 53% of cases (*n* = 17).^19^ Other clinical staff reported that patients were likely to refuse blood tests 43% (*n* = 13).^19^ Tungaraza & Farooq^22^ reported that 54% (*n* = 99) of practicing psychiatrists felt that likely poor adherence to the drug was a reason for delays.^22^

### Health system-related barriers

Studies based on clinician surveys identified the following health system-related barriers.

(a) Difficulties in obtaining baseline bloods and the time taken to register patients for blood monitoring were considered as major factors in initiating clozapine by 22% (*n* = 26) clinicians.^17^
(b) Staff resources, including inadequate staff to monitor clozapine initiation, were a major factor for 22% (*n* = 26) of clinicians in delaying clozapine initiation.^17^
(c) The need for admission as required by some health providers to initiate clozapine and a shortage of beds were highlighted by 20% (*n* = 23) of clinical staff.^17^ In another survey, 32% (*n* = 40) of clinical staff felt that a lack of resources in the home treatment team led to frequent delays in commencing clozapine.^17^
(d) Service fragmentation owing to separate teams providing community and in-patient services and a lack of community support were cited as major barriers (*n* = 39) by clinicians in one study.^22^ A survey of staff at Ashworth high-secure hospital also revealed that clinicians felt that a lack of resources was responsible for delays or non-prescription of clozapine in 16% (*n* = 5) of cases.^19^

Stroup *et al*^25^ conducted a retrospective study using Medicaid claims data from 45 states in the USA. It was found that among 629 809 unique antipsychotic treatment episodes, 79 934 showed service use patterns consistent with treatment resistance. Clozapine accounted for 2.5% of starts of antipsychotic medication among patients in the overall sample, and 5.5% of starts among patients with treatment resistance. Clozapine initiation was significantly associated with male sex, younger age, White ethnicity, more frequent out-patient service use for schizophrenia, and greater prior-year hospital use for mental health.^25^ Patients residing in counties with historically high clozapine usage were almost twice as likely to start clozapine as patients residing in historically low-use counties (adjusted odds ratio (AOR) 2.03; CI 1.75–2.30).^25^ A high concentration of psychiatrists (>15 per 100 000 residents) was also associated with a greater likelihood of clozapine initiation (AOR 1.17; CI 1.03–1.33).^25^ However, there were no significant effects of population density or measures of poverty or income on clozapine initiation.

Goren *et al*^26^ carried out 70 semi-structured interviews with stakeholders such as psychiatrists, mental health nurses, and pharmacy and laboratory staff at five sites with high clozapine use and five low-utilization sites. Low utilization of clozapine was associated with a lack of champions to support the clozapine process. Some of the barriers highlighted included the complex and time-consuming paperwork.^26^ Lack of transport, particularly for rural patients, inability by disorganised patients to navigate public transport, paranoia around travelling by public transport and the cost of transportation^26^ were reported as major barriers. Patients living far away from clinics were not considered suitable for clozapine owing to their inability to attend for regular blood tests.^26^

Kelly *et al*^18^ elicited the views of psychiatrists using an anonymous survey questionnaire. The questionnaire consisted of 56 questions to be scored on a five-point Likert scale (1 = strongly disagree, 5 = strongly agree) regarding the barriers related to clozapine, and the physician's interest and willingness to use point-of-care (POC) devices. The survey was sent to 860 psychiatrists, of whom 277 (32%) responded. Among the 28 listed barriers (clinical, nonclinical, and side-effects) to more frequent use of clozapine, the two highest ranking barriers were: (a) the likely non-adherence of patients to blood work (score 3.7 ± 1.1) and (b) the burden of ongoing blood monitoring for the patient (score 3.6 ± 1.2). Among nine potential solutions for increasing the use of clozapine, the use of POC devices was the highest ranked. The physicians agreed that a POC device would improve care and that it would increase their clozapine use with a mean score of 3.9 ± 1.0.^18^

The National Association of State Mental Health Program Directors (NASMHPD) in in the USA formed a working group to identify barriers to clozapine underutilization and interventions to overcome these at a national level.^27^ The initial work group included 11 clinicians and researchers and consulted a wide range of stakeholders and existing literature on the subject. They identified 14 major barriers, which included all the factors mentioned above, as well as benign ethnic neutropenia (BEN), which occurs among people of African or Middle Eastern ancestry. The lack of a definition for BEN in product labelling and clear guidance on monitoring requirements may be responsible for the low use of clozapine in this population. A lack of standardised materials for shared decision-making, complex protocols for treatment monitoring and management of side-effects, formulary issues and costs of ancillary services such as transportation and service coordination were also identified as barriers (Box 1).^27^

Box 1 Barriers to clozapine use and strategies to overcome these

Barriers to clozapine use

Patient/drug-related barriers

• Refusal of blood tests^17^^–^^19^
• Tolerating clozapine and side-effects^17^
• Age > 20^19^^,^^20^
• Patients prescribed polypharmacy^21^
• Benign ethnic neutropenia^27^

Clinician-related barriers

• Inadequate knowledge of or experience in clozapine use^17^^,^^22^^–^^24^
• Fear of side-effects for patient/lack of knowledge about clozapine side-effects^13^^,^^17^^,^^19^^,^^22^^,^^24^
• Lack of adherence to guidance^13^^,^^17^^,^^22^
• Difficulty identifying suitable patients and unclear diagnoses^22^
• Need for intense monitoring^22^^,^^24^
• Perception that patients may not comply with treatment^19^^,^^22^

Health system-related barriers

• Not enough resources, including not enough staff resources to monitor clozapine inititation^17^
• Shortage of beds^17^
• Service fragmentation^21^
• Lack of champions to support the clozapine process^26^
• Complex and time-consuming paperwork^26^
• Lack of standardised shared decision-making^27^
• Complex protocols for treatment monitoring^27^
• Formulary issues and costs of ancillary services such as transport and service coordination^27^

Strategies to overcome barriers to clozapine use

• Use of POC devices^29^
• Support for prescribers and decision-aid tool for consumers grounded in principles of shared decision-making^27^
• Internet-based educational programmes to provide information for consumers, family members and clincians^27^

#### Interventions to overcome the barriers

Three studies described interventions that could help to overcome the barriers identified above. These included a training initiative^28^ and two studies describing the use of POC devices.^28^^,^^29^ As these studies employed different methodologies and interventions, the results are briefly summarised here.

Bogers *et al*^29^ compared a POC device using capillary blood sampling with a finger prick that provided WBC counts with conventional venous sampling. An open-label randomised cross-over trial design was used to compare the two procedures. The main outcome measure was the subjective experience of various aspects of blood sampling, as measured by a visual analogue scale (VAS). A consistent pattern in favour of capillary blood sampling was found (total perceived burden blood sampling: capillary 5.79 *v.* venous 13.4 (*P* < 0.001)). Both patients and practitioners showed preferences for the capillary blood sampling.^29^

Similarly, Nielsen *et al*^30^ evaluated a POC using a randomised cross-over trial design. Patients were randomised to one of two blood monitoring sequences. The first group underwent venous sampling followed by capillary sampling in a twice-repeated procedure (venous–capillary–venous–capillary); in the other, the sequence was reversed (capillary–venous–capillary–venous). Eighty-five patients were included in the study using a VAS; patients indicated that they found capillary blood monitoring less painful than venous sampling (VAS ratings: 0.55 cm 25–75th percentiles: 0.1–1.4 cm *v.*. 1.75 cm 25–75th percentiles: 0.7–2.6, *P* < 0.001). They also felt less inconvenienced by the POC method than by traditional blood sampling.^30^

Carruthers *et al*^28^ described an educational intervention to promote the evidence-based use of clozapine in New York,^28^ consisting of support for the prescriber and a decision aid tool for consumers grounded in the principle of shared decision-making. A manual for clinicians was developed and academics presented a series of state-wide grand rounds presentations to provide information on clozapine prescribing. Internet-based educational programmes and a telephone consultation service by experts to support the prescribers were also provided. A key component of the programme was testimonials from patients, who described personal benefits alongside the challenges.^28^ The programme was evaluated using Medicaid data on the pattern of new antipsychotic start-ups. The number of new starts amongst all antipsychotic trials increased from 1.5% in 2009 to 2.1% 2013.^28^ The greatest change was seen in state-operated facilities, where the rate of clozapine new starts per quarter increased compared with all new antipsychotic starts. The change in the rate of clozapine new starts in these facilities was three times higher than in other settings (3.77% *v.* 1.13%).^28^

#### Quality assessment of included studies

The published protocol outlined separate quality assessments for qualitative and quantitative studies, using appropriate checklists for different study designs.^15^ However, after examining the included studies, it was felt that only two trials^29^^,^^30^ could be assessed for quality using the risk of bias tool, as per protocol. These randomised cross-over trials compared capillary blood sampling using a POC device with traditional venous blood sampling. Patients were randomised to two sequences, starting with either capillary or venous blood sampling, followed by a repeated sequence. Neither of these trials provided details of how participants were randomly allocated to the two sequences, and the outcome assessments did not appear to have been done by blind assessors. Both studies had high risk of bias.

Other studies did not use appropriate study designs, which could be evaluated using the checklists we proposed in the protocol for observational studies. These studies were mostly surveys and provided little information on how the samples were selected and the validity or reliability of the questionnaires/instruments used, or any information on non-responders. All these studies were considered to be of low quality.

## Discussion

This was the first systematic review aiming to examine the barriers to effective use of clozapine. The following major barriers or factors related were identified: the mandatory blood testing requirement; fear of serious side-effects, lack of familiarity in use of clozapine; lack of clarity in diagnosis and difficulty in identifying suitable patients; service fragmentation; and lack of adequate training in or exposure to using clozapine. Only one educational intervention was available that showed some effect on clozapine prescription rate. POC testing using capillary blood was more acceptable to patients than traditional blood sampling, being less painful and less time consuming, but no studies tested whether it increased the uptake of clozapine.

A conservative estimate suggests that TRS adds more than $34 billion in annual direct medical costs in the USA.^31^ In the UK, NICE has included the extent and the degree of clozapine use in the quality criteria for commissioners when commissioning services for mental health.^32^ However, initiatives to overcome this major service need are rare.

Almost all studies highlighted routine blood monitoring as the top-ranking barrier to initiating and maintaining clozapine treatment. Two randomised cross-over trials showed that blood testing using a simple finger prick that was undertaken as part of routine assessment by psychiatric staff, either in the patient's home or at a psychiatric out-patient clinic, was feasible and convenient for patients. However, none of these trials looked at the effect of POC testing on prescription rates. The POC devices will also need to comply with regulatory requirements for monitoring blood counts.

It appears that there is a common perception amongst clinicians that clozapine is a dangerous drug, and that patients will not adhere to it or would not like to consider it as a treatment option. The findings in this review suggest that these negative beliefs about clozapine result from a lack of experience and knowledge, owing to the current limited use of clozapine. A self-perpetuating cycle can ensue, as practitioners do not see the benefits of clozapine, and thus do not develop confidence in its use.^1^ This is consistent with the study by Stroup *et al* which showed that higher clozapine initiation was significantly associated with patients residing in areas associated with historically high clozapine usage and higher concentrations of psychiatrists (>15 per 100 000 population).^25^

The NASMHPD published 36 recommendations on its website for expanding the use of clozapine.^33^ One important recommendation included improving residency trainee standards. Considering the disease burden resulting from TRS and the central role of clozapine in its treatment, we suggest that training in the use of clozapine becomes a mandatory requirement for all psychiatry residence and continuing professional development programmes. A certification requiring competence in initiating, maintaining and managing side-effects of clozapine is required, based on clinical experience, similar to the certification that is now required for electroconvulsive therapy.

The use of clozapine is alarmingly low in many developing countries. In Pakistan, for example, about 1300 patients were receiving clozapine as recorded in the Clozaril Patient Monitoring System. Although generic clozapine has become available recently, numbers are still very low, considering that the country has a population of about 200 million (R. U. Rahman, personal communication, 2016; data available from the authors on request). To put this prescription rate into perspective, The Netherlands, with a population of about 17 million, has over 12000 patients on clozapine, which is 0.07% of the population (https://www.gipdatabank.nl/). This means that, at current rates of use, there is a more than 100-fold difference between the two countries. This situation requires a public health intervention to improve access to clozapine in certain countries.

The major limitation of the review was the low quality of the included studies. Studies were based on surveys, which are prone to a number of biases, including selection bias of respondents, and lacked control groups. The low numbers of studies from a few countries also limit the generalisation of results. None of the studies defined the optimal use of clozapine. The lack of patient perspectives is striking, considering that a number of studies suggested patient-related factors as major barriers.

Despite these limitations, this systematic review indicates that there is broad agreement on the major barriers that hinder the effective use of clozapine. There is certainly a need to improve the methodological quality of studies and the way these are reported, but the present study identifies gaps in clinical practice and health services that can be addressed in intervention studies. Use of POC devices, educational interventions targeting clinicians and shared decision-making involving patients need to be evaluated using controlled study designs. Future research should be guided by the implementation science methods and behaviour change principles that have successfully been used in implementing and evaluating evidence-based interventions in medicine.

## About the authors

**Saeed Farooq** is a clinical senior lecturer at the Research Institute for Primary Care & Health Sciences, Keele University, a visiting professor at the University of Chester and Honorary Consultant Psychiatrist, Midlands Partnership NHS Foundation Trust, UK. **Abid Choudry** is an ST6 at Forward Thinking Birmingham, Birmingham Women's and Children's NHS Foundation Trust, UK. **Dan Cohen** is a psychiatrist at the Department of Community Mental Health, Mental Health Organization North-Holland NorthHeerhugowaard, The Netherlands. **Farooq Naeem** is a professor at the University of Toronto and a staff psychiatrist at the Centre for Addiction and Mental Health, Toronto, Canada. **Muhammad Ayub** is a professor and Chair of the Division of Developmental Disabilities at the Department of Psychiatry, Queen's University, Kingston, Canada.
